# Retrospective Study on Genetic Diversity and Drug Resistance among People Living with HIV at an AIDS Clinic in Beijing

**DOI:** 10.3390/ph17010115

**Published:** 2024-01-16

**Authors:** Yan-Ze Shi, Hui-Huang Huang, Xin-Hua Wang, Bing Song, Tian-Jun Jiang, Min-Rui Yu, Ze-Rui Wang, Rui-Ting Li, Yan-Mei Jiao, Xin Su, Fu-Sheng Wang

**Affiliations:** 1 Medical School of Chinese People’s Liberation Army (PLA), Beijing 100853, China; shiyanze@126.com (Y.-Z.S.); ymr121331@163.com (M.-R.Y.); wzr301gh@163.com (Z.-R.W.); 2 Department of Infectious Diseases, The Fifth Medical Centre of Chinese PLA General Hospital, National Clinical Research Centre for Infectious Diseases, Beijing 100853, China; hhh302@163.com (H.-H.H.); 302wangxh@sina.com (X.-H.W.); aba302@163.com (T.-J.J.); 3 Department of Gastroenterology, First Medical Center of Chinese PLA General Hospital, Beijing 100036, China; 4 State Key Laboratory of Pathogenand Biosecurity, Beijing Institute of Microbiology and Epidemiology, Academy of Military Medical Sciences (AMMS), Beijing 100850, China; kurui1991@163.com

**Keywords:** HIV, pretreatment drug resistance, acquired drug resistance, Beijing

## Abstract

(1) Background: The objective of this study was to investigate the prevalence of genetic diversity and drug resistance mutations among people living with HIV (PLWH) attending clinics in Beijing. (2) Methods: A retrospective analysis was conducted on PLWH admitted to the Fifth Medical Center of People’s Liberation Army (PLA) General Hospital between 1 March 2013 and 31 July 2020. The participants were analyzed for pretreatment drug resistance (PDR) and acquired drug resistance (ADR). Nested polymerase chain reaction (PCR) was utilized to amplify the pol gene from plasma RNA samples obtained from the participants. Genotypic and HIV drug resistance were determined using the Stanford University HIV Drug Resistance Database. Univariate and multifactorial logistic analyses were used to assess the risk factors for PDR. (3) Results: The overall prevalence rates of PDR and ADR were 12.9% and 27.8%, respectively. Individuals treated with non-nucleoside reverse transcriptase inhibitors (NNRTIs) exhibited the highest prevalence of mutations. Specific mutation sites, such as V179D for NNRTIs and M184V and K65R for nucleoside reverse transcriptase inhibitors (NRTIs), were identified as prevalent mutations. Individuals treated with efavirenz (EFV) and nevirapine (NVP) were found to be susceptible to developing resistance. The multifactorial regression analyses indicated that the factors of circulating recombination form (CRF) genotype CRF07-BC and a high viral load were associated with an increased risk of PDR. CRF01-AE and CRF07-BC were the most prevalent HIV genotypes in our study. (4) Conclusions: The distribution of HIV genotypes in Beijing is complex. There is a need for baseline screening for HIV drug resistance among ART-naive individuals, as well as timely testing for drug resistance among ART-experienced individuals.

## 1. Introduction

HIV infection is a significant health issue with implications for public health. While effective antiretroviral therapy (ART) can reduce HIV-related illnesses and mortality, it needs to be continued indefinitely, as people living with HIV require lifelong medication. With the increasing number of individuals on treatment, HIV drug resistance (HIVDR) testing and knowledge of drug resistance have become increasingly important. HIVDR testing can be performed using either phenotypic or genotypic assays. Phenotypic assays provide valuable data but are costly and time-consuming, hence their limited use nowadays [1,2,3,4]. On the other hand, genotypic assays are more cost-effective, provide faster results, and are the most commonly used routine tests due to their ability to detect wild-type and drug-resistant virus mixtures with higher sensitivity.

HIV drug resistance can be divided into primary and acquired drug resistance (ADR). Primary resistance can be further subdivided into pretreatment drug resistance (PDR) and transmitted drug resistance (TDR) [5]. TDR has been previously studied in treatment-naive populations with no history of exposure. PDR is a broader term that encompasses TDR and resistance to initiation of or re-entry into ART and is more commonly used to describe situations where the duration of infection is unknown, and it is uncertain whether it is TDR [6]. The prevalence of PDR poses challenges for HIV epidemic control and vaccine development [7], and it has important implications for selecting first-line ART in key populations, such as men who have sex with men (MSM), sex workers, injecting drug users, transgender individuals, and incarcerated individuals. It may also contribute to potential reasons for treatment failure within these groups [8]. ADR occurs under drug stress and severely impairs the efficacy of drugs, posing a significant risk to HIV treatment. Furthermore, it could lead to future HIV epidemics driven by drug-resistant strains [9].

The prevalence of PDR and ADR in China varies significantly depending on factors such as study design, geographic location, and economic and social backgrounds [5,10]. In China, the overall level of PDR was reported to be 5.56% [11]. However, certain areas showed slightly higher prevalence rates, such as Liangshan Prefecture in Sichuan Province (12.2%) [12], Pu’er in Yunnan Province (10.8%) [13], Guangxi Province (8.3%) [14], and Hunan Province (10.0%) [15]. Major cities like Shanghai (17.4%) [16], Xi’an (18.3%) [17], and Chongqing (10.54%) [18] had higher prevalence rates of PDR due to the complexity and diversity of genotypes, with an increasing trend overall [5]. In terms of ADR, the overall level in China was reported to be 51.33% [11]. There were significant regional differences in prevalence rates, specifically for Shanghai (69.3%) [19], Hefei (38.6%) [20], Henan (64.76%) [21], and Liaoning (64.84%) [22]. Overall, the central region of China had a higher prevalence of ADR (72.44%) than other regions (north: 47.92%; south: 50.59%) [11]. It is worth noting that there are differences between China’s ART program and those of other countries. China started highly active antiretroviral therapy (HAART) at the end of 1999 [23], and the National Free Antiretroviral Treatment Program (NFATO) was implemented in 2002 [24]. Initially, the available drug options were limited, such as zidovudine (AZT), stavudine (D4T), didanosine (DDI), nevirapine (NVP), etc., and the first-line treatment regimen was often AZT/D4T + DDI + NVP [25]. However, some drugs had side effects and high rates of drug resistance. As new drugs have been introduced since, the optimal ART regimen has changed frequently, gradually shifting towards using two nucleoside reverse transcriptase inhibitors (NRTIs) combined with a third drug class, which can be non-nucleoside reverse transcriptase inhibitors (NNRTIs), boosted protease inhibitors (PIs), or integrase inhibitors (INSTIs). Single-tablet regimens (STRs) have also become an option [26]. However, the extent of drug resistance increases with the duration of treatment, so timely HIV drug resistance testing is essential [27,28,29].

So far, long-term studies on PDR and ADR in Beijing have not been reported. The Fifth Medical Center of People’s Liberation Army (PLA) General Hospital is a specialized center for HIV prevention and treatment which maintains a stable cohort for HIV follow-up [30,31]. In this study, we utilized our follow-up cohort to examine drug resistance among ART-naive individuals and those experiencing virologic failure. The objectives of this study were to evaluate drug resistance and the distribution of genotypes and to generate data on the long-term prevalence of HIV drug resistance in Beijing.

## 2. Results

### 2.1. Demographic and Clinical Characteristics

**Figure 1:**
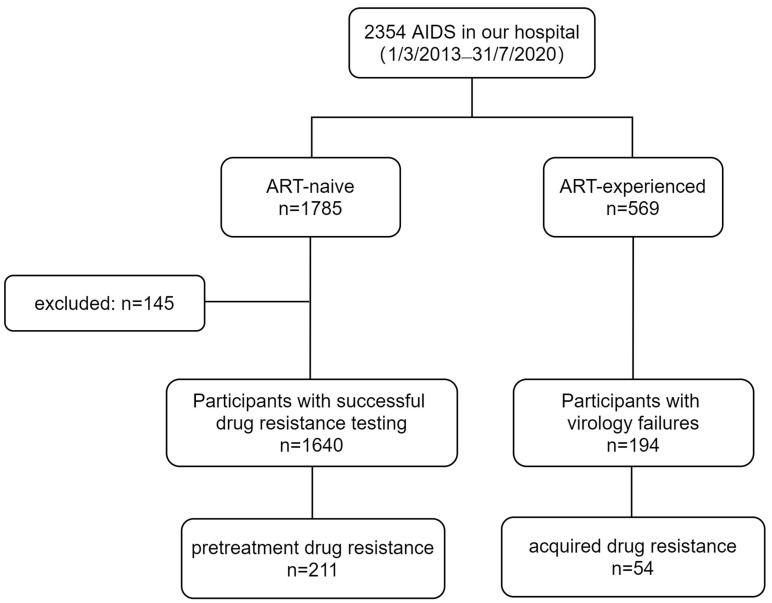
Composition of the treatment-naive cohort and follow-up cohort at the Fifth Medical Center of PLA General Hospital for the period 2013–2020.

A total of 1785 ART-naive and 569 ART-experienced individuals were enrolled in this study (Figure 1). After excluding 145 ART-naive individuals without sequence results, the final analysis included 1640 ART-naive individuals for PDR analysis and 569 ART-experienced individuals for ADR analysis. Among the 1640 ART-naive individuals, the median age was 31 years (interquartile range (IQR) 26–40). The majority of participants were male (97.0%, 1590/1640), while a smaller proportion were female (3.5%, 50/1640). The primary mode of transmission was male homosexual transmission (89.2%, 1463/1640). The median CD4 count was 292 cells/µL (IQR 175–422), and the median HIV viral load was 4.7 log10 copies/mL (IQR 4.3–5.3) (Table 1).

Among the 569 ART-experienced individuals, the median age was 30 years (IQR 26–41); the median CD4 count was 318 cells/µL (IQR 142–421); and the median HIV viral load was 4.8 log10 copies/mL (IQR 4.5–5.3). The majority of participants were male (97.7%, 556/569), and the main route of transmission was male homosexual transmission (89.6%, 510/569). In total, 99.5% (566/569) of participants underwent therapy including a combination of two NRTIs with one NNRTI, while 0.5% (3/569) used a combination of two NRTIs with PIs (Table 1).

### 2.2. Genotype Distribution

**Figure 2:**
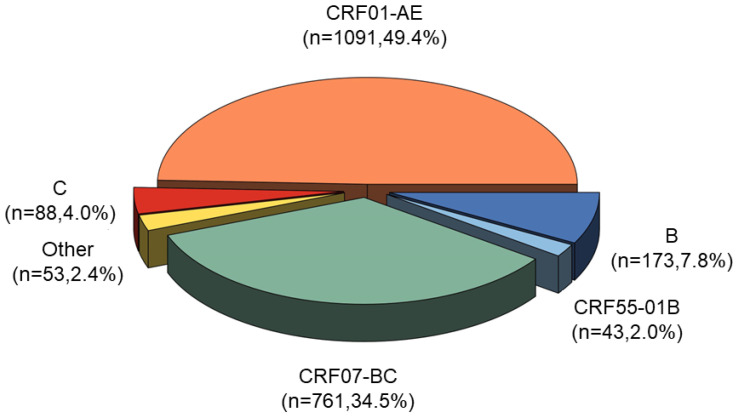
Genotype distribution among all participants.

The distribution of genotypes in this study is presented in Figure 2. Among the 2209 participants, we identified a total of five major genotypes and several other genotypes. The circulating recombination forms (CRFs) CRF01-AE (49.4%, 1091/2209) and CRF07-BC (34.5%, 761/2209) were the most prevalent genotypes, followed by B (7.8%, 173/2209), C (4.0%, 88/2209), CRF5501-B (2.0%, 43/2209), and others (2.4%, 53/2209).

### 2.3. Pretreatment Drug Resistance Mutations

**Figure 3:**
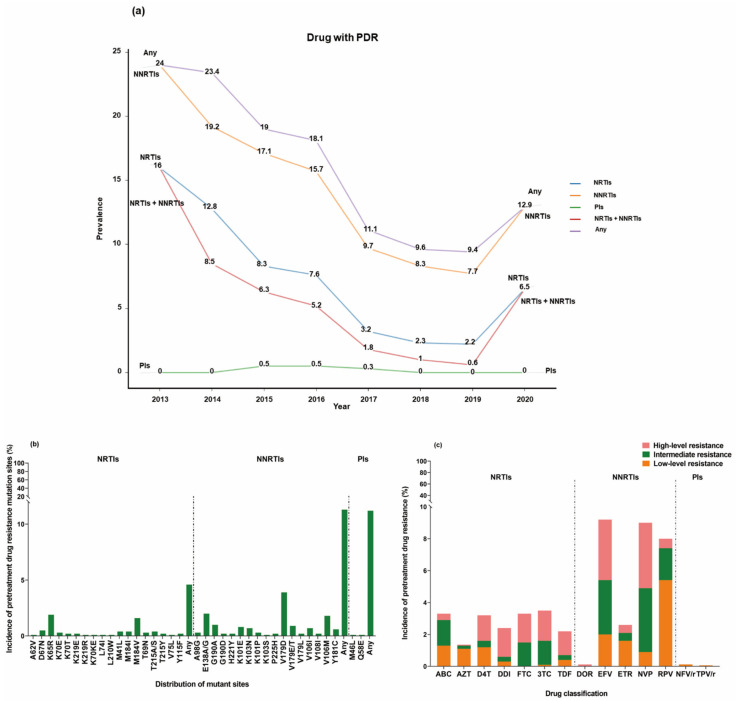
The data in the graphs are differentiated by antiretroviral drug class. “Any” refers to mutations resistant to any of the drugs. (**a**) Temporal trends in the annual proportion of pretreatment drug resistance mutations among ART-naive participants overall. (**b**) Percentage and level of susceptibility to pretreatment resistance mutations. (**c**) Distribution of sites with pretreatment drug resistance mutations.

Among the ART-naive individuals, 211 participants (12.9%, 211/1640) displayed pretreatment resistance mutations. The overall prevalence of PDR mutations was 12.9% (211/1640). The highest prevalence of drug resistance mutations was observed in individuals treated with NNRTIs (11.2%, 184/1640), followed by those treated with NRTIs (4.6%, 75/1640) and PIs (0.2%, 3/1640), and all of them showed a decreasing trend until 2019 (Figure 3a). Among the NNRTI resistance mutations, the most common associated mutation was V179D (3.9%, 64/1640), followed by E138A/G (2.0%, 33/1640), V106M (1.8%, 29/1640), G190A (1.0%, 17/1640), and V179E/T (0.9%, 14/1640). NRTI resistance-associated mutations were less prevalent, with K65R (1.9%, 31/1640) and M184V (1.6%, 26/1640) being the most frequent ones. M46L (0.1%, 2/1640) and Q58E (0.1%, 1/1640) were the major sites associated with resistance to PIs (Figure 3b).

In order to analyze drug resistance to commonly used antiretroviral (ARV) drugs in clinical settings, we tested a total of 15 ARV drugs, which covered the most frequently used first- and second-line drugs. Our findings showed high-level resistance in 151 ART-naive individuals (9.2%, 151/1640). Among NNRTI resistance mutations, the highest proportion of resistance mutations was observed for efavirenz (EFV) (9.2%, 151/1640), followed by NVP (9.0%, 148/1640) and rilpivirine (RPV) (8.3%, 136/1640). Most variants (10.3%, 169/1640) exhibited susceptibility to low-level drug resistance. However, high-level resistance was predominantly observed for EFV- and NVP-treated individuals (3.8%, 63/1640), with 3.2% (53/1640) showing high-level resistance to both EFV and NVP. Regarding NRTIs, less than 1.0% of NRTIs were associated with drug resistance mutations, but all of them were intermediate- or high-level mutations. The most frequently observed resistance mutations were found for lamivudine (3TC) (3.5%, 58/1640), followed by abacavir (ABC) (3.3%, 54/1640), emtricitabine (FTC) (3.3%, 54/1640), and D4T (3.2%, 52/1640). Only three participants had resistance mutations against PIs. Among them, Nelfinavir/r (NFV/r) (0.1%, 2/1640) and Tipranavir/r (TPV/r) (0.1%, 1/1640) were both susceptible to low-level resistance (Figure 3c).

The overall prevalence of PDR in our study was 12.9%, which falls within the mid-range level. This is consistent with the results of studies conducted in Sichuan [12], Pu’er of Yunnan [13], Hunan [15], and Chongqing [18]. When compared to some major cities in China [16,17] and foreign studies [6], our prevalence of PDR was still relatively low. The highest incidence of resistance was observed for NNRTI, with V179D and K65R being the predominant resistance mutations. These findings align with studies conducted in Tianjin [32] and Cameroon [33]. It has been widely reported that EFV and NVP, as first-line drugs, are frequently associated with the development of resistance [33,34].

### 2.4. Drug Resistance Mutations in ART-Experienced Participants

**Figure 4:**
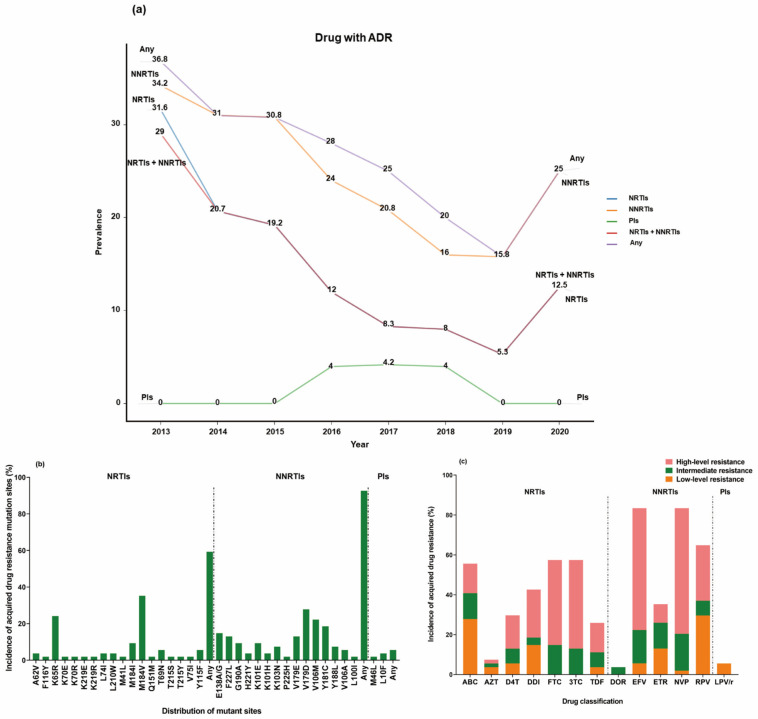
(**a**) Temporal trends in the annual proportion of antiretroviral drug resistance mutations among ART-experienced participants overall. (**b**) Distribution of sites in individuals with drug resistance mutation who experienced virologic failure. (**c**) Percentage and level of susceptibility to acquired drug resistance mutations.

Among the 569 ART-experienced individuals, there were 194 participants who experienced virologic failure, and 54 of them (27.8%, 54/194) exhibited acquired resistance mutations. The prevalence of mutations resistant to NNRTIs, NRTIs, and PIs were 92.6% (50/54), 59.3% (32/54), and 5.6% (3/54), respectively. Out of the 54 participants, 57.4% (31/54) showed mutations resistant to both NRTIs and NNRTIs, while 5.6% (3/54) had mutations resistant to PIs; the highest prevalence was that of mutations resistant to NNRTIs, with an overall decreasing trend until 2019 (Figure 4a). The most common sites associated with mutations resistant to nNRTIs, NRTIs, and PIs were V179D (27.8%, 15/54), M184V (35.2%, 19/54), and L10F (3.7%, 2/54), respectively (Figure 4b).

Among the participants with ADR mutations, the following drugs were associated with a high level of resistance: NVP (63.0%, 34/54), EFV (61.1%, 33/54), 3TC (44.4%, 24/54), FTC (42.6%, 23/54), and DDI (24.1%, 13/54). No intermediate-level nor high-level drug resistance to PIs was observed (Figure 4c). Among the participants, 23 individuals (42.6%) showed high-level resistance to both FTC and 3TC, while 33 individuals (61.1%) exhibited high-level resistance to both EFV and NVP. Additionally, 21 individuals (38.9%) displayed high-level resistance to NVP, EFV, 3TC, and FTC.

Unlike previous studies, our study observed an overall prevalence of ADR at 27.8%, which is significantly lower than the rates reported in Xi’an [17], Guangdong [35], and Portugal [36,37]. Consistent with the findings of numerous studies, EFV and NVP were found to be the most susceptible to drug resistance [35,36].

### 2.5. Factors Associated with Pretreatment Drug Resistance in ART-Naive Individuals

The results of the multifactorial regression analysis revealed that heterosexual transmission (OR 1.78, 95% CI 1.00–3.15; *p* = 0.05) and unknown routes of infection (OR 2.62, 95% CI 1.23–5.58; *p* = 0.01) were associated with an increased risk of PDR mutations. CRF07-BC (OR 1.85, 95% CI 1.34–2.55; *p* < 0.001) was also significantly associated with an increased risk of PDR mutations. Furthermore, viral load (OR 2.01, 95% CI 1.39–2.90; *p* < 0.001) showed a positive association with the prevalence of PDR mutations. However, other variables, such as age, sex, and CD4 count, did not show significant associations with PDR mutations (Table 2).

## 3. Discussion

This study provides a systematic analysis of HIV drug resistance over an eight-year period at an acquired immunodeficiency syndrome (AIDS) clinical center in Beijing, and its results are consistent with the findings of other studies on HIV drug resistance in Beijing [38,39]. The overall prevalence of PDR in our study was 12.9%, with a significant downward trend from 2015 to 2019, which might be related to the policy of immediate treatment upon detection implemented in China after 2015. The prevalence of ADR was lower than that reported in other areas, which may be related to good compliance among our participants and the strict management system. These findings are consistent with those of several retrospective studies conducted in China [40,41]. The prevalence rate of PDR in our study was lower than those reported in Tianjin (13.5%) [32], Shanghai (17.4%) [16], Yunnan (34.2%) [13], and Guangxi (21.2%) [14]. This difference may be due to variations in the follow-up and management of PLWH in different regions. Additionally, the presence of multiple AIDS centers in Beijing compared with other cities may contribute to better access to healthcare and medication for PLWH in Beijing.

The prevalence of PDR was the highest for NNRTIs among the three types of antiretroviral drugs (Figure 3a). Among the mutation sites, 30.3% (64/211) were clustered on V179D, which was associated with potentially high-level resistance to NNRTIs. Mutations in V179D/E can lead to a 2–5 times reduction in susceptibility to drugs like NVP, EFV, ETR, and RPV [42]. Our study also observed similar results, with V179D causing high and intermediate levels of resistance to NVP and EFV and low-level resistance to RPV, EFV, NVP, and ETR. Although V179D/E mutations do not appear to significantly impact virologic response to first-line regimens, their high prevalence (3.9%, 64/1640) suggests caution in regimen selection. The HIV treatment guidelines recommend replacing the NNRTI with lopinavir/r (LPV/r) for second-line regimens. PIs mainly showed low-level resistance to NFV/r and TPV/r, consistent with previous studies [39], suggesting that PIs are viable options for second-line regimens following the baseline regimen. Based on our findings, the prevalence of PDR mutations in ART-naive individuals in Beijing was significantly higher for NNRTIs (87.2%, 184/211) than for NRTIs (35.5%, 75/211) and PIs (1.4%, 3/211), underscoring the importance of immediate PDR testing after diagnosis. According to the World Health Organization’s guidelines for updated public health response to PDR, countries with NNRTI PDR mutation rates exceeding 10% should consider changing their first-line ART regimens from NNRTIs to non-NNRTI drugs, including INSTIs. Therefore, our findings suggest that physicians should make informed decisions in selecting appropriate treatment regimens.

The primary cause of virologic failure among ART-experienced individuals was found to be ADR mutations. The prevalence of ADR mutations for NNRTIs was 92.6% (50/54), which was significantly higher than that for NRTIs (59.3%, 32/54) and PIs (5.6%, 3/54). The most prevalent mutation site for NRTIs was M184V, which may have been influenced by the frequent use of 3TC in Beijing [43,44]. This was followed by K65R, which is strongly associated with high-level resistance to FTC, DDI, D4T, and TDF. We also observed that the presence of both M184V and K65R mutations could lead to reduced treatment effectiveness in regimens containing ABC, DDI, FTC, and 3TC, in agreement with findings of a study on HIV resistance among MSM in southern China [43]. Due to the limited number of participants using PIs in our study, further research with an expanded sample size is needed to investigate the prevalence of ADR mutations for PIs.

We identified six common CRFs and multiple other genotypes in our study. The dominant genotypes were CRF01-AE and CRF07-BC, which is consistent with previous findings on the distribution of HIV genotypes in China [45]. Due to the high population mobility and the complex and diverse HIV genotypes in Beijing, we also detected some uncommon genotypes in our study. For instance, there was a relatively small proportion of CRF65-cpx (0.6%, 13/2209) with a high prevalence of pretreatment resistance (4.3%, 9/211). These findings highlight the importance of monitoring the sources and modes of transmission for genotypes with a high prevalence of drug resistance in order to prevent the spread of complex drug-resistant genotypes. The prevalence of genotype C in Beijing was low, with only nine individuals showing pretreatment resistance to genotype C, mainly involving non-nucleotide inhibitors. Another study conducted in Beijing identified that 33% of infected individuals with genotype C exhibited resistance mutations for NNRTIs, which was significantly associated with pretreatment resistance [39]. Although genotypes B and C are not the predominant strains in Beijing, their relatively high prevalence of pretreatment resistance to NNRTIs suggests that physicians should be vigilant regarding this resistance pattern. Furthermore, the E138 mutation shows high prevalence in CRF08-BC, and this specific mutation pattern mainly confers moderate- or low-level resistance to RPV but not to EFV, NVP, doravirine (DOR), or etravirine (ETR) [9]. In our study, CRF08-BC strains were susceptible to resistance mutations at the E138A site, as well as V179E and P225H, and exhibited intermediate levels of resistance to both EFV and NVP, as well as low levels of resistance to RPV. Therefore, PLWH who carry the CRF08-BC genotype should consider alternative regimens that do not include EFV, RPV, or NVP.

Multifactorial analysis revealed that the CRF07-BC genotype was associated with an increased risk of PDR mutations. This finding aligns with a prospective multicenter study conducted in China [46], which demonstrated an association between the CRF07-BC genotype and the prevalence of drug resistance mutations at baseline. Additionally, we found a positive correlation between a high viral load and PDR, consistent with a European multicenter study [47], suggesting the importance of regular viral load monitoring. In contrast to some previous studies [32], gender (specifically the female gender) was not identified as a risk factor in our study. This could be attributed to the small number of women included in our study, which made it difficult to specifically assess the effect of gender on PDR.

## 4. Materials and Methods

### 4.1. Study Design and Data Collection

We conducted a retrospective analysis of PDR and ADR in all PLWH at PLA General Hospital’s Fifth Medical Center from 1 March 2013 to 31 July 2020. The inclusion criteria for this study were as follows: (1) complete general information, (2) ART-naive or ART-experienced status prior to enrollment, and (3) agreement to undergo drug resistance testing. The study was approved by the Ethics Committee of the Fifth Medical Center of PLA General Hospital. Demographic information (gender, age) and epidemiological information (route of transmission, time of diagnosis, initiation of ART, current regimen) were collected retrospectively (Table 1). The plasma was separated within 6 h of blood collection and stored at −80 °C for subsequent use. A total of 1785 ART-naive individuals and 569 ART-experienced individuals were enrolled in this study. PDR analysis was performed on all ART-naive participants, while ADR analysis was conducted on those with ART duration exceeding 6 months (Figure 1).

### 4.2. Sample Collection, Laboratory Tests, and Definitions

Peripheral blood specimens were collected for viral load and HIV drug resistance testing. CD4 counts were measured using flow cytometry (FACS Caliber; BD Biosciences, Franklin Lakes, NJ, USA). HIV RNA levels were quantified using polymerase chain reaction (PCR) (COBAS amplification/TaqMan; Roche, Porterville, CA, USA), and a viral load of <20 copies/mL was considered undetectable.

### 4.3. RNA Extraction and Sanger Sequencing

HIV RNA was extracted from 500 μL of plasma using a QIAamp Viral RNA Mini kit (QIAGEN, Hilden, Germany). Fragments of the HIV *pol* gene, including protease and partial reverse transcriptase (RT) genes, were amplified using an in-house nested PCR method. The amplified fragments were then sent to Biomed Co. (Beijing, China) for sequencing.

### 4.4. Genotyping and Drug Resistance Analysis

HIV genotyping was performed using Rega HIV Genotype Analysis Tool 3.0 [48]. Resistance mutations were analyzed using the Stanford HIV Resistance Database (Stanford HIVdb; version 8.8) [49]. These mutations were classified as low-, intermediate-, or high-level resistance mutations. The level of resistance to a specific drug was determined by summing the penalty scores associated with each drug resistance mutation present in the sequence. The total score determined the estimated level of resistance to the drug, which was categorized into the following five categories: susceptible (total score of 0 to 9), potential low-level resistance (total score of 10 to 14), low-level resistance (total score of 15 to 29), intermediate-level resistance (total score of 30 to 59), and high-level resistance (total score ≥ 60). In this study, all low-, intermediate-, and high-level resistance mutations were considered drug-resistant mutations.

### 4.5. Statistical Analysis

Statistical analyses were conducted using SPSS 26.0 (IBM SPSS Statistics for Windows, IBM Corp., Armonk, New York, NY, USA). Non-normally distributed measures were described using medians and interquartile ranges (IQRs), while categorical variables were presented as percentages. Categorical variables were analyzed with the chi-squared test or Fisher’s exact test. Potential risk factors associated with PDR were selected for univariate logistic regression analysis. Variables with *p*-values < 0.05 were included in the multivariate logistic regression model. *p*-values < 0.05 were considered statistically significant.

## 5. Conclusions

In conclusion, this study presents long-term data on the prevalence trends of pretreatment drug resistance and virologic failure due to drug resistance in Beijing. The overall trend shows a decrease in HIV drug resistance mutations. However, it is important to note that the COVID-19 pandemic has had an impact, leading to disruptions in the scheduled treatment and follow-up of PLWH. As a result, the number of individuals included in the study for 2020 was reduced. These findings highlight the need for increased attention to the management of special populations, such as PLWH, and emphasize the importance of epidemic prevention and control measures in preventing the serious consequences of drug resistance.

## Figures and Tables

**Table 1:** Demographic and clinical characteristics of participants.

Characteristics	ART-Naive Individuals (*n* = 1640)	ART-Experienced Individuals (*n* = 569)
Gender		
Male	1590 (97.0)	556 (97.7)
Female	50 (3.5)	13 (2.3)
Age (years)	31 (26, 40)	30 (26, 41)
<30	704 (42.9%)	248 (43.6)
30–49	752 (45.9%)	245 (43.1)
≥50	184 (11.2%)	76 (13.4)
Route of transmission		
Homosexual	1463 (89.2)	510 (89.6)
Heterosexual	115 (7.0)	34 (6.0)
Other	20 (1.2)	10 (1.8)
Unknown	42 (2.6)	14 (2.5)
CD4 count (cells/μL)	292 (175, 422)	318 (142, 421)
Viral load (log10copies/mL)	4.7 (4.3, 5.3)	4.8 (4.5, 5.3)
Duration of ART (years)		
<1	_	414 (72.8)
1–5	_	122 (21.4)
≥5	_	33 (5.8)
ART regimen		
NRTIs + NNRTIs	_	566 (99.5)
NRTIs + PIs	_	3 (0.5)

Note: Data are expressed as *n* (%) or medians (interquartile ranges). Abbreviations: _, not applicable.

**Table 2:** Factors associated with drug resistance mutations among ART-naive participants.

	Univariate Analysis			Multivariable Analysis		
Variables	OR	95% CI	*p*	OR	95% CI	*p*
Gender						
Male	Ref.	Ref.	Ref.	Ref.	Ref.	Ref.
Famale	1.19	(0.59–2.42)	0.63	0.74	(0.30–1.82)	0.51
Age (years)						
<30	Ref.	Ref.	Ref.	Ref.	Ref.	Ref.
30–49	0.88	(0.55–1.42)	0.60	1.12	(0.82–1.53)	0.47
≥50	1.08	(0.81–1.44)	0.58	0.68	(0.40–1.15)	0.15
Route of transmission						
Homosexual	Ref.	Ref.	Ref.	Ref.	Ref.	Ref.
Heterosexual	1.89	(1.00–3.57)	0.05	1.78	(1.00–3.15)	0.05
Other	0.75	(0.19–3.04)	0.69	0.69	(0.15–3.08)	0.62
Unknow	1.50	(1.00–2.33)	0.07	2.62	(1.23–5.58)	0.01
HIV subtype						
CRF01-AE	Ref.	Ref.	Ref.	Ref.	Ref.	Ref.
CRF07-BC	1.80	(1.31–2.47)	<0.001	1.85	(1.34–2.55)	<0.001
B	1.11	(0.60–2.07)	0.74	1.19	(0.63–2.23)	0.59
C	1.52	(0.75–3.09)	0.25	1.81	(0.87–3.77)	0.11
CRF55-01B	1.86	(0.75–4.61)	0.18	2.01	(0.80–5.07)	0.14
Other	1.71	(0.57–5.10)	0.34	2.05	(0.68–6.21)	0.20
CD4 count (cell/μL)						
<200	Ref.	Ref.	Ref.	Ref.	Ref.	Ref.
200–499	0.70	(0.47–1.04)	0.08	1.39	(0.96–1.99)	0.08
≥500	1.00	(0.73–1.37)	0.99	1.10	(0.72–1.70)	0.65
Viral load (copies/mL)						
≤10,000	Ref.	Ref.	Ref.	Ref.	Ref.	Ref.
10,001–100,000	0.88	(0.60–1.28)	0.49	0.85	(0.58–1.25)	0.41
≥100,001	1.91	(1.35–2.71)	<0.001	2.01	(1.39–2.90)	<0.001

Abbreviations: OR, odds ratio; CI, confidence interval.

## Data Availability

The data used to support the findings of this study are available from the corresponding author upon reasonable request.

## References

[1] Petropoulos C.J., Parkin N.T., Limoli K.L., Lie Y.S., Wrin T., Huang W., Tian H., Smith D., Winslow G.A., Capon D.J. (2000). A novel phenotypic drug susceptibility assay for human immunodeficiency virus type 1. Antimicrob. Agents Chemother..

[2] Walter H., Schmidt B., Korn K., Vandamme A.M., Harrer T., Uberla K. (1999). Rapid, phenotypic HIV-1 drug sensitivity assay for protease and reverse transcriptase inhibitors. J. Clin. Virol..

[3] Cane P. (2011). HIV drug resistance testing. Methods Mol. Biol..

[4] Metzner K.J. (2022). Technologies for HIV-1 drug resistance testing: Inventory and needs. Curr. Opin. HIV AIDS.

[5] Yuan D., Liu Y., Zhou Y., Shi L., Chen J., Lu J., Fu G., Wang B. (2022). Men who have sex with men is the high-risk drug resistance population: A meta-analysis of HIV-1 drug resistance profiles and trends in China. J. Clin. Pharm. Ther..

[6] Trebelcock W.L., Lama J.R., Duerr A., Sanchez H., Cabello R., Gilada T., Segura P., Reisner S.L., Mayer K.H., Mullins J. (2019). HIV pretreatment drug resistance among cisgender MSM and transgender women from Lima, Peru. J. Int. AIDS Soc..

[7] Zeng R., Ren D., Gong X., Wei M., Gao L., Yu A., Zhang D., Mi Y., Ma P. (2020). HIV-1 Genetic Diversity and High Prevalence of Pretreatment Drug Resistance in Tianjin, China. AIDS Res. Hum. Retroviruses.

[8] Macdonald V., Mbuagbaw L., Jordan M.R., Mathers B., Jay S., Baggaley R., Verster A., Bertagnolio S. (2020). Prevalence of pretreatment HIV drug resistance in key populations: A systematic review and meta-analysis. J. Int. AIDS Soc..

[9] Tchouwa G.F., Eymard-Duvernay S., Cournil A., Lamare N., Serrano L., Butel C., Bertagnolio S., Mpoudi-Ngole E., Raizes E., Aghokeng A.F. (2018). Nationwide Estimates of Viral Load Suppression and Acquired HIV Drug Resistance in Cameroon. eClinicalMedicine.

[10] Guo P., Lan Y., Li Q., Ling X., Li J., Tang X., Hu F., Cai W., Li L. (2022). Pre-treatment Drug Resistance Could Impact the 96-Week Antiretroviral Efficacy in Treatment-Naive HIV-1–Infected Patients in Guangdong, China. Infect. Dis. Immun..

[11] Kang R.H., Liang S.J., Ma Y.L., Liang S., Xiao L., Zhang X.H., Lu H.Y., Xu X.Q., Luo S.B., Sun X.G. (2020). Pretreatment HIV drug resistance in adults initiating antiretroviral therapy in China, 2017. Infect. Dis. Poverty.

[12] Li D., Chen H., Li H., Ma Y., Dong L., Dai J., Jin X., Yang M., Zeng Z., Sun P. (2022). HIV-1 pretreatment drug resistance and genetic transmission network in the southwest border region of China. BMC Infect. Dis..

[13] Chen M., Zhu Q., Xing H., Chen H., Jin X., Dong L., Dai J., Yang M., Yang C., Jia M. (2020). The characteristics of pretreatment HIV-1 drug resistance in western Yunnan, China. Epidemiol. Infect..

[14] Zhang F., Liang B., Liang X., Lin Z., Yang Y., Liang N., Yang Y., Liang H., Jiang J., Huang J. (2021). Using Molecular Transmission Networks to Reveal the Epidemic of Pretreatment HIV-1 Drug Resistance in Guangxi, China. Front. Genet..

[15] Cao X., Cao J., Qi H., Yu W., Zeng Z., Peng Y., Wang M. (2023). Prevalence of Primary Drug Resistance Among Newly Diagnosed HIV-1-Infected Individuals in Hunan Province, China. AIDS Res. Hum. Retroviruses.

[16] Wang Z., Zhang M., Zhang R., Liu L., Shen Y., Wang J., Lu H. (2019). Diversity of HIV-1 genotypes and high prevalence of pretreatment drug resistance in newly diagnosed HIV-infected patients in Shanghai, China. BMC Infect. Dis..

[17] Xia H., Jin J., Ba H., Zhang Y., Li J., Guo R., Li Y., Ma P., Zhang Y. (2023). Genetic Diversity and Characteristics of Drug Resistance Among Treatment-Naive People Living with HIV in Xi’an, China. Drug Des. Devel Ther..

[18] Liu M., He X.Q., Deng R.N., Tang S.Q., Harypursat V., Lu Y.Q., He K., Huo Q., Yang H.H., Liu Q. (2022). Pretreatment drug resistance in people living with HIV: A large retrospective cohort study in Chongqing, China. HIV Med..

[19] Zhang F., Liu L., Sun M., Sun J., Lu H. (2017). An analysis of drug resistance among people living with HIV/AIDS in Shanghai, China. PLoS ONE.

[20] Zheng S., Wu J., Hao J., Wang D., Hu Z., Liu L., Song C., Hu J., Lei Y., Wang H. (2022). Epidemic Characteristics of HIV Drug Resistance in Hefei, Anhui Province. Pathogens.

[21] Liu J., Wu Y., Yang W., Xue X., Sun G., Liu C., Tian S., Sun D., Zhu Q., Wang Z. (2015). Population-based human immunodeficiency virus 1 drug resistance profiles among individuals who experienced virological failure to first-line antiretroviral therapy in Henan, China during 2010–2011. AIDS Res. Ther..

[22] Wei Q., Zhao Y., Lv Y., Kang X., Pan S., Yao S., Wang L. (2022). High Rate of HIV-1 Drug Resistance in Antiretroviral Therapy-Failure Patients in Liaoning Province, China. AIDS Res. Hum. Retroviruses.

[23] Luo L., Li T.S. (2011). Overview of antiretroviral treatment in China: Advancement and challenges. Chin. Med. J..

[24] Ding Y., Ma Z., He J., Xu X., Qiao S., Xu L., Shi R., Xu X., Zhu B., Li J. (2019). Evolving HIV Epidemiology in Mainland China: 2009–2018. Curr. HIV/AIDS Rep..

[25] Cao W., Hsieh E., Li T. (2020). Optimizing Treatment for Adults with HIV/AIDS in China: Successes over Two Decades and Remaining Challenges. Curr. HIV/AIDS Rep..

[26] AIDS and Hepatitis C Professional Group, Society of Infectious Diseases, Chinese Medical Association, Chinese Center for Disease Control and Prevention (2021). Chinese guidelines for diagnosis and treatment of HIV/AIDS (2021 ed.). Zhonghua Nei Ke Za Zhi.

[27] Stadeli K.M., Richman D.D. (2013). Rates of emergence of HIV drug resistance in resource-limited settings: A systematic review. Antivir. Ther..

[28] Gupta R.K., Jordan M.R., Sultan B.J., Hill A., Davis D.H., Gregson J., Sawyer A.W., Hamers R.L., Ndembi N., Pillay D. (2012). Global trends in antiretroviral resistance in treatment-naive individuals with HIV after rollout of antiretroviral treatment in resource-limited settings: A global collaborative study and meta-regression analysis. Lancet.

[29] Weng Y.W., Chen I.T., Tsai H.C., Wu K.S., Tseng Y.T., Sy C.L., Chen J.K., Lee S.S., Chen Y.S. (2019). Trend of HIV transmitted drug resistance before and after implementation of HAART regimen restriction in the treatment of HIV-1 infected patients in southern Taiwan. BMC Infect. Dis..

[30] Zhang L.X., Jiao Y.M., Zhang C., Song J.W., Fan X., Xu R.N., Huang H.H., Zhang J.Y., Wang L.F., Zhou C.B. (2020). HIV Reservoir Decay and CD4 Recovery Associated With High CD8 Counts in Immune Restored Patients on Long-Term ART. Front. Immunol..

[31] Zhang L.X., Song J.W., Zhang C., Fan X., Huang H.H., Xu R.N., Liu J.Y., Zhang J.Y., Wang L.F., Zhou C.B. (2021). Dynamics of HIV reservoir decay and naïve CD4 T-cell recovery between immune non-responders and complete responders on long-term antiretroviral treatment. Clin. Immunol..

[32] Gao L., Xia H., Zeng R., Wu Y., Zaongo S.D., Hu Y., Ma P. (2022). Pre-treatment and acquired antiretroviral drug resistance among people living with HIV in Tianjin, China. HIV Med..

[33] Fokam J., Chenwi C.A., Tala V., Takou D., Santoro M.M., Teto G., Dambaya B., Anubodem F., Semengue E.N.J., Beloumou G. (2023). Pre-Treatment HIV Drug Resistance and Genetic Diversity in Cameroon: Implications for First-Line Regimens. Viruses.

[34] García-Morales C., Tapia-Trejo D., Matías-Florentino M., Quiroz-Morales V.S., Dávila-Conn V., Beristain-Barreda Á., Cárdenas-Sandoval M., Becerril-Rodríguez M., Iracheta-Hernández P., Macías-González I. (2021). HIV Pretreatment Drug Resistance Trends in Mexico City, 2017-2020. Pathogens.

[35] Lan Y., Xin R., Cai W., Deng X., Li L., Li F., Cai X., Tang X., Fan Q., Hu F. (2020). Characteristics of drug resistance in HIV-1 CRF55_01B from ART-experienced patients in Guangdong, China. J. Antimicrob. Chemother..

[36] Pingarilho M., Pimentel V., Diogo I., Fernandes S., Miranda M., Pineda-Pena A., Libin P., Theys K., Martins M.R.O., Vandamme A.M. (2020). Increasing Prevalence of HIV-1 Transmitted Drug Resistance in Portugal: Implications for First Line Treatment Recommendations. Viruses.

[37] Fokam J., Chenwi C.A., Takou D., Santoro M.M., Tala V., Teto G., Beloumou G., Semengue E.N.J., Dambaya B., Djupsa S. (2023). Laboratory Based Surveillance of HIV-1 Acquired Drug Resistance in Cameroon: Implications for Use of Tenofovir-Lamivudine-Dolutegravir (TLD) as Second- or Third-Line Regimens. Viruses.

[38] Ye J., Hao M., Xing H., Wang Y., Wang J., Feng Y., Xin R., Zeng J., Zhao S., Hao Y. (2020). Characterization of subtypes and transmitted drug resistance strains of HIV among Beijing residents between 2001-2016. PLoS ONE.

[39] Song Y.X., Xin R.L., Li Z.C., Yu H.W., Lun W.H., Ye J., Liu A., Li A.X., Li J.W., Ye J.Z. (2018). Prevalence of transmitted drug resistance among HIV-1 treatment-naive patients in Beijing. Epidemiol. Infect..

[40] Dong K., Ye L., Leng Y., Liang S., Feng L., Yang H., Su L., Li Y., Baloch S., He F. (2019). Prevalence of HIV-1 Drug Resistance among Patients with Antiretroviral Therapy Failure in Sichuan, China, 2010-2016. Tohoku J. Exp. Med..

[41] Liu P., Liao L., Xu W., Yan J., Zuo Z., Leng X., Wang J., Kan W., You Y., Xing H. (2018). Adherence, virological outcome, and drug resistance in Chinese HIV patients receiving first-line antiretroviral therapy from 2011 to 2015. Medicine.

[42] Rhee S.Y., Gonzales M.J., Kantor R., Betts B.J., Ravela J., Shafer R.W. (2003). Human immunodeficiency virus reverse transcriptase and protease sequence database. Nucleic Acids Res..

[43] Lan Y., Deng X., Li L., Cai W., Li J., Cai X., Li F., Hu F., Lei C., Tang X. (2021). HIV-1 Drug Resistance and Genetic Transmission Networks Among MSM Failing Antiretroviral Therapy in South China 2014-2019. Infect. Drug Resist..

[44] Vannappagari V., Ragone L., Henegar C., van Wyk J., Brown D., Demarest J., Quercia R., St Clair M., Underwood M., Gatell J.M. (2019). Prevalence of pretreatment and acquired HIV-1 mutations associated with resistance to lamivudine or rilpivirine: A systematic review. Antivir. Ther..

[45] Xiao P., Li J., Fu G., Zhou Y., Huan X., Yang H. (2017). Geographic Distribution and Temporal Trends of HIV-1 Subtypes through Heterosexual Transmission in China: A Systematic Review and Meta-Analysis. Int. J. Environ. Res. Public Health.

[46] Li Y., Gu L., Han Y., Xie J., Wang H., Lv W., Song X., Li Y., Iwamoto A., Ishida T. (2015). HIV-1 subtype B/B’ and baseline drug resistance mutation are associated with virologic failure: A multicenter cohort study in China. J. Acquir. Immune Defic. Syndr..

[47] Prosperi M.C., Mackie N., Di Giambenedetto S., Zazzi M., Camacho R., Fanti I., Torti C., Sönnerborg A., Kaiser R., Codoñer F.M. (2011). Detection of drug resistance mutations at low plasma HIV-1 RNA load in a European multicentre cohort study. J. Antimicrob. Chemother..

[48] de Oliveira T., Deforche K., Cassol S., Salminen M., Paraskevis D., Seebregts C., Snoeck J., van Rensburg E.J., Wensing A.M., van de Vijver D.A. (2005). An automated genotyping system for analysis of HIV-1 and other microbial sequences. Bioinformatics.

[49] Siepel A.C., Halpern A.L., Macken C., Korber B.T. (1995). A computer program designed to screen rapidly for HIV type 1 intersubtype recombinant sequences. AIDS Res. Hum. Retroviruses.

